# From intestinal colonization to systemic infections: *Candida albicans* translocation and dissemination

**DOI:** 10.1080/19490976.2022.2154548

**Published:** 2022-12-11

**Authors:** Jakob L. Sprague, Lydia Kasper, Bernhard Hube

**Affiliations:** a Department of Microbial Pathogenicity Mechanisms, Leibniz Institute for Natural Product Research and Infection Biology - Hans Knöll Institute, Jena, Germany; b Institute of Microbiology, Friedrich Schiller University, Jena, Germany

**Keywords:** *Candida albicans*, intestinal translocation, dissemination, fungi, gut colonization

## Abstract

*Candida* species are the most prevalent cause of invasive fungal infections, of which *Candida albicans* is the most common. Translocation across the epithelial barrier into the bloodstream by intestinal-colonizing *C. albicans* cells serves as the main source for systemic infections. Understanding the fungal mechanisms behind this process will give valuable insights on how to prevent such infections and keep *C. albicans* in the commensal state in patients with predisposing conditions. This review will focus on recent developments in characterizing fungal translocation mechanisms, compare what we know about enteric bacterial pathogens with *C. albicans*, and discuss the different proposed hypotheses for how *C. albicans* enters and disseminates through the bloodstream immediately following translocation.

## 1. Introduction

Species within the *Candida* genus are the most prevalent cause of invasive fungal infections. Such *Candida* infections that reach the bloodstream, termed candidemia, can occur in patients with a variety of predisposing medical conditions, from gastrointestinal surgery to immunosuppression.^1,2^ Once in the bloodstream, *Candida* spp. can disseminate and colonize other organs, such as the spleen and kidneys.^1,2^ The opportunistic pathogen *Candida albicans* accounts for most candidemia cases.^2,3^ *C. albicans* is known to reside as a commensal in the human gastrointestinal (GI) tract and several studies propose that the GI tract population serves as a source for systemic infections.^4^ For example, it was shown that intestinal colonizing strains were identical with strains isolated from the bloodstream prior to systemic infection.^5,6^ A review of data from studies available at the time concluded that an endogenous source of infection from the gut, as opposed to an external source, was the most likely and well supported.^7^ It was recently observed that systemic infection of various *Candida* spp. was preceded by expansion of a single *Candida* species in the GI tract of hematopoietic stem cell transplant patients. The intestinal isolates were identical to those later isolated from the bloodstream.^8^ The expansion of *C. parapsilosis* in the GI tract was also associated with a decrease in the total number of bacteria, while domination by *C. albicans* was associated with decreased bacterial diversity.^8,9^ These data all suggest that the GI tract likely functions as the main source for disseminated candidiasis in humans. The process from intestinal colonization, crossing the epithelial barrier, and subsequently colonizing other organs *via* the bloodstream has been described using various *in vivo* murine models of endogenously disseminated candidiasis.^10–15^ While colonization of the GI tract by *C. albicans* and host–cell interactions during the early stages of infection have been extensively studied *in vitro* and discussed,^16–19^ the later stages of translocation across the gut epithelial barrier and dissemination *via* the bloodstream remain more undefined. Here, we will review what is known about commensal growth of *C. albicans* during intestinal colonization, how *C. albicans* translocates from the GI tract into the bloodstream, and how this differs from what is already known for more well-studied bacteria. We will further discuss the current open questions regarding *C. albicans* translocation and dissemination.

## 2. Intestinal colonization phenotype

**Figure 1.**
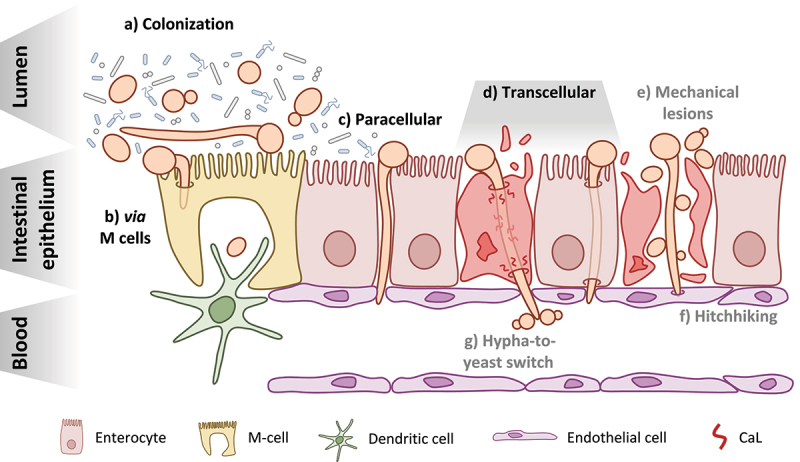
*Candida albicans* at the intestinal barrier: from colonization to translocation and dissemination. a) *C. albicans* can be found in both the yeast and hyphal morphotypes during colonization of the GI tract of healthy individuals and must compete with other members of the microbiota b) Translocation *via* M cells present in intestinal lymphoid tissues like Peyer’s patches may occur following either induced endocytosis or active penetration by *C. albicans* and may also involve the fungus hijacking the sampling function of resident phagocytes, like dendritic cells c) The paracellular route of translocation refers to invasion of the intestinal barrier by *C. albicans* hyphae in the spaces between enterocytes without actually invading the host cells d) The transcellular route of translocation could occur in two manners: with candidalysin (CaL)-dependent damage of the epithelial cells resulting in necrotic cell death (left) or in the absence of host-cell damage (right) This appears to be the major route used by *C. albicans* to cross the intestinal barrier e) One hypothesis for how yeast reach the bloodstream is that they are able to move across the epithelial barrier from the GI tract to the bloodstream *via* physical disruptions in the tissue, caused either by the fungus itself or some other factors like surgery or chemotherapy f) The so-called “hitchhiking” hypothesis posits that yeast present in the GI tract may move through the barrier along with invading hyphae by attaching to them as they invade the epithelium g) Finally, the hypha-to-yeast switch hypothesizes that translocating hyphae undergo a morphological transition once reaching the bloodstream that initiates the formation of yeast cells from the hyphae.

A fundamental aspect of *C. albicans* that sets it apart from other intestinal colonizers, like bacteria, and even other fungi is its morphological plasticity (Figure 1a). *C. albicans* morphology is influenced by many environmental factors, such as body temperature, physiological pH and the presence of certain amino acids among others.^20^ The ability of *C. albicans* to freely transition from yeast to hyphal morphologies greatly contributes to its success as a commensal colonizer and opportunistic pathogen.^21,22^ There is a large body of evidence that the yeast morphology favors commensal colonization of the GI tract of antibiotic-treated and germ-free mice, though both morphologies are present.^23–26^ In fact, evolution of *C. albicans* within the murine GI tract selects for hypha-defective mutant strains.^27^ Passage through the GI tract can even induce a specialized yeast cell type called GUT cells *via* the transcription factor *WOR1*. These cells exhibit a shift in their metabolism toward nutrients that are prevalent in the GI tract.^28^

Hypha formation and genes expressed during filamentation can also trigger negative selection from the host. Loss of the major hyphal regulator genes *EFG1, ROB1, BRG1*, or *TEC1* during competition in a murine model increased colonization of *C. albicans*.^24^ *Vice versa*, increased filamentation of *C. albicans via* loss of a negative regulator for hypha formation significantly decreased colonization in an antibiotic-treated mouse model.^29^ Similarly, loss of the hyphal regulator genes *ZCF8, ZFU2*, and *TRY4* led to hyper-filamentation and decreased colonization of the GI tract of germ-free mice.^25^ In the same study, Böhm and colleagues^25^ also found that overexpression of *UME6* had a detrimental effect on intestinal colonization. *UME6* is responsible for hyphal extension during growth in filamentation-inducing conditions.^30^ While overexpression of *UME6* reduced competitive fitness in the GI tract of antibiotic-treated mice, loss of the gene conferred a competitive advantage and allowed a deletion mutant strain to outcompete the WT during intestinal colonization.^24,25^ Interestingly, the *in vivo* ratio of yeast to hyphae in the gut of a Ume6-deficient strain was similar to that of the wild-type strain.^30^ This increased competitive fitness was instead due to a loss of the secreted protease Sap6 and the surface adhesin Hyr1, suggesting that hypha-associated factors rather than the hyphal morphology *per se* are detrimental for colonization in antibiotic-treated mice.^24^

Within a healthy host, *C. albicans* must cope with competition with other microorganisms and a variety of host factors, with a potential impact on fungal morphology (Figure 1a). The host seems able to actively select for the yeast morphology during colonization *via* the adaptive immune system. Intestinal IgA specifically targets hypha-specific adhesins to select against hyphae and may actually increase the competitive fitness of *C. albicans* yeast cells during intestinal colonization.^31,32^ Additionally, *C. albicans* colonization of the murine GI tract induces the production of anti-fungal IgG that protects against systemic infection.^33^ This IgG pool also prevents fungal translocation from the GI tract, thereby helping to keep *C. albicans* in a commensal state.^33^ Intestinal colonization with *C. albicans* also induces anti-fungal Th-17 cells that protect against systemic infection and are even cross-reactive against other fungal pathogens; however, it has not yet been shown whether such Th-17 responses directly influence *C. albicans* morphology or translocation from the GI tract into the bloodstream.^34,35^ The role that these adaptive immune responses play in colonization and infection of *C. albicans* has been recently reviewed.^36,37^

Recent studies suggest that the microbiome composition and level of bacterial colonization are key factors that determine the degree of *C. albicans* colonization in mice and humans.^9,26,38^ In fact, most mouse models rely on removal of gut bacteria by antibiotic treatment to allow for stable intestinal colonization of *C. albicans* lab strains.^15,39,40^ Without antibiotic treatment, antagonistic effects of gut microbiota on fungal growth or morphology may limit *C. albicans* colonization. Some *Lactobacillus* species, for example, have been shown to antagonize *C. albicans* pathogenicity on intestinal epithelial cells as well as interfere with hyphal elongation *via* a variety of mechanisms, such as production of short chain fatty acids.^41–44^ *Lactobacillus rhamnosus* can even induce a hypha-to-yeast switch in *C. albicans* through production of various metabolites when cultured with enterocytes, thereby reducing the fungus’ ability to cause damage.^42^ A similar inhibition of hypha formation has been observed in the presence of *Enterococcus faecalis*.^43^ The extent to which such antagonistic effects affect fungal colonization *in vivo*, is not fully understood. In fact, colonization by *C. albicans* is also possible without antibiotics, though to a lesser degree.^45^ In addition, there is conflicting evidence whether or not laboratory mice are naturally resistant to colonization with *C. albicans*.^46,47^ Recent studies showed increased fungal colonization when using rewilded mice or human *C. albicans* isolates instead of standard laboratory strains.^26,38^ Diet alterations have also been shown to facilitate stable *C. albicans* gut colonization in mice even without antibiotic treatment, possibly due to changes in the bacterial microbiome.^48^

While there are many factors which seem to promote yeast growth in the GI tract during colonization, this does not appear to be sufficient to completely suppress hyphal growth (Figure 1a). Though they showed a competitive advantage for strains lacking major hyphal regulators, Witchley and colleagues^24^ observed yeast and hyphae present in all gut compartments of antibiotic-treated mice, matching results from other studies.^25,26^ They also found increased expression of hypha-associated genes in the gut compared to the yeast inoculum, such as *ECE1, ALS3* and *HWP1*, similarly to a previous study.^24,49^ The transition from yeast to hyphal growth has long been considered one of the most important virulence factors for *C. albicans*. This process of filamentation plays a vital role during *C. albicans* infection and has been discussed as a promising target for antifungal drugs.^21,22^ In fact, the previously mentioned hypha-associated genes (*ECE1, ALS3*, and *HWP1*) are some of the most well-characterized virulence factors for *C. albicans* and also part of the core filamentation response that comprises commonly upregulated genes under a variety of filament-inducing conditions.^50,51^ Whether hyphae play a distinct role during colonization or filamentation simply cannot be fully suppressed by the host and microbiota remains to be determined. However, since the majority of *C. albicans* isolates have kept the potential to form hyphae and the commensal stage is the predominant lifestyle of *C. albicans*, there must be a selective advantage of hypha formation during colonization.

Furthermore, the presence of hyphae in the GI tract prior to infection may provide some benefit to *C. albicans* as it transitions from commensal to pathogen. In the following sections, we will discuss the stages of infection immediately following colonization of the gut, dissect the mechanisms involved, and how these morphotypes contribute to each step.

## 3. Translocation across the intestinal epithelium

As previously mentioned, many predisposing factors increase the risk of translocation of *C. albicans* from the GI tract to the bloodstream. Trauma, intestinal surgery, chemotherapy, and immunosuppression can all lead to candidemia.^1,2^ Many host factors are critical for preventing fungal translocation from the gut, including a balanced gut microbiome, intact intestinal barrier and cellular immunity, particularly neutrophils.^15,45^ The contribution of surgery and physical disruption of the intestinal barrier will be discussed in section 4.2, but here we will focus on *C. albicans*-mediated translocation and the strategies the fungus uses to overcome the intestinal epithelium. The interaction of *C. albicans* with the host at mucosal barriers has been recently reviewed with detailed descriptions of the different players (gut microbiota, intestinal epithelial cells, and gut mucosal immunity) as well as the fungal factors involved and models that can be used to study such processes.^19^ Here, we focus on the major translocation routes observed or proposed for *C. albicans* (transcellular, *via* microfold cells, and paracellular), compare the knowledge on fungal translocation to known mechanisms used by bacteria, and highlight the current knowledge gaps concerning fungal translocation.

### 3.1 Transcellular translocation

**Figure 2.**
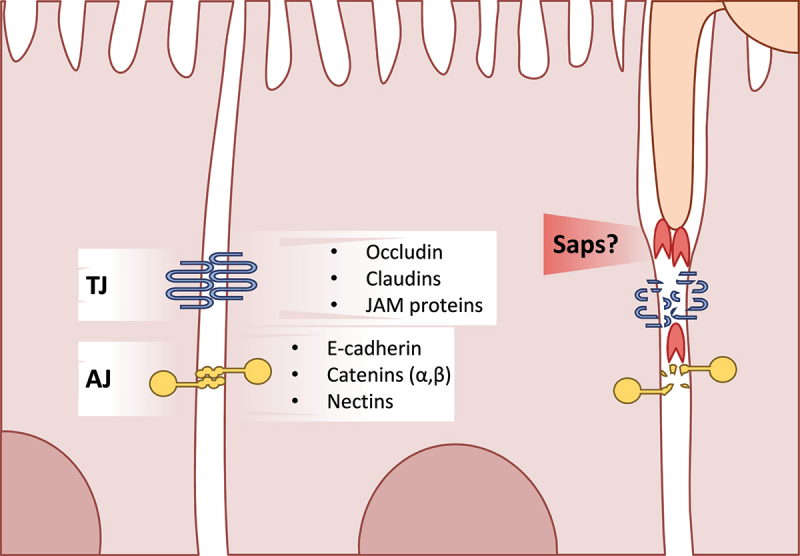
A glance at intestinal epithelial cell junctions. An overview of the major components of cell-cell junctions of enterocytes Tight junctions (TJ) are mainly comprised of the occludin, claudin, and junction adhesion molecule (JAM) proteins Adherens junctions (AJ) in intestinal epithelial cells are mostly comprised of E-cadherin, catenins, and nectins *C. albicans* is capable of degrading tight and adherens junction proteins, potentially *via* secretion of Sap5.

Translocation across the intestinal epithelium *via* non-phagocytic cells, referred to as transcytosis, is well characterized for many bacteria both *in vitro* and *in vivo*.^52^ *Salmonella typhimurium* induces actin rearrangements and endocytosis by the direct injection of the SopE, SopE2, and SopB effector proteins *via* its type III secretion system.^53^ *Listeria monocytogenes* specifically binds E-cadherin on the surface of enterocytes to induce internalization and subsequent exocytosis.^54^ This is similar to the process of induced endocytosis observed during infection of oral epithelial cells *in vitro* with *C. albicans*. During the early stages of infection, adhesion of *C. albicans* to oral epithelial cells triggers actin-dependent, induced endocytosis *via* binding of the fungal adhesin Als3 to host E-cadherin.^55,56^ This process can also be observed during infection of intestinal epithelial cells *in vitro*, though only with alteration of tight junction proteins between host cells (Figure 2). Induced endocytosis of *C. albicans* by enterocytes was observed after widening of the intercellular spaces with patulin treatment.^57^ This study also shows that intact and mature tight junctions of the intestinal epithelium inhibit induced endocytosis by *C. albicans*, limiting invasion to active penetration by hyphae.

The major route for invasion of intestinal epithelial cells by *C. albicans* in *in vitro* models is active penetration.^56^ This suggests that the main mode of translocation through the intestinal epithelium likely involves filamentation, penetration and damage of host cells, in contrast to the more host-driven mechanisms of bacterial transcytosis. Damage of epithelial cells is driven by the *ECE1* gene, which encodes the peptide toxin candidalysin (CaL).^58^ CaL-mediated damage was shown to be required for the efficient translocation of *C. albicans* across enterocytes.^59^ The main route appeared to be transcellular in this system as efficient translocation was associated with necrotic cell death and required the full damage potential of invading hyphae (Figure 1d). However, a recent study showed that *C. albicans* invasion of enterocytes occurs largely in the absence of any apparent disruption of the host membrane for up to 9 hours of infection.^60^ Though host-cell damage and death were observed for invasion events after 20 hours,^59,60^ this supports the hypothesis that transcellular translocation is possible through enterocytes in the absence of significant host damage (Figure 1d). This might also explain the low levels of translocation seen for the non-damaging *C. albicans* strain lacking *ECE1*, as it exhibits normal hypha formation and invasion of epithelial cells.^58,59^ Translocation was also dependent on hypha formation as filamentation-deficient strains lacking both *EFG1* and *CPH1* also showed a decreased rate of translocation.^59^ This is further supported by the observation that *L. rhamnosus*, which protects intestinal epithelial cells from *C. albicans* damage and limits filamentation, also significantly decreased fungal translocation.^41^

In contrast to the more host-driven process of induced endocytosis that is utilized by bacteria, *C. albicans* translocation through enterocytes seems to be mainly dependent on hypha formation, invasion, and host damage. While we know that both yeast and hyphae are present in the GI tract during colonization, the roles these morphologies play during intestinal translocation *in vivo* remain unknown. The contribution of colonization-induced host responses during fungal translocation of *C. albicans* is also largely unknown as most studies focus on *in vitro* model systems, which lack any host immune system. Further studies are required to elucidate, for example, whether yeast cells are able to translocate themselves or together with hyphae and if the colonizing hyphae are sufficient to initiate translocation or if there is an expansion of hyphae preceding translocation.

### 3.2 Translocation via microfold cells and Peyer’s patches

The contributions of microfold (M) cells and Peyer’s patches of the intestine to translocation of bacterial pathogens during systemic infection has been well studied and reviewed.^52^ M cells are specialized intestinal epithelial cells for phagocytosis of material from the lumen and its presentation to resident phagocytes, like dendritic cells (Figure 1b).^61^ M cells are located within lymphoid tissues of the gut, like Peyer’s patches in the small intestine.^61^ Bacteria such as *Shigella flexneri* and *S. typhimurium* are known to specifically target M cells during infection. *S. flexneri* can also enter M cells, though not exclusively, and use these as a mechanism for translocation across the epithelium.^62^ *S. typhimurium* exclusively invades M cells causing extensive host-cell death and is even able to enter adjacent epithelial cells.^63^ *S. typhimurium* has also been shown to use phagocytic cells present at Peyer’s patches for transport into the bloodstream. The presence of dendritic cells in an *in vitro* model with intestinal epithelial cells facilitated transport across the barrier.^64^ The same study also showed the uptake of *S. typhimurium* from the intestine in an *in vivo* mouse model.

In contrast to bacteria, the involvement of M cells during translocation of *C. albicans* across the intestinal epithelium has not been fully elucidated. It is known that *C. albicans*, along with *C. tropicalis*, are sampled by Peyer’s patches and taken into lymphoid tissues in a partially M cell-dependent manner *in vivo* (Figure 1b).^65^ *C. albicans* was also shown to preferentially invade M cells in a mixed, *in vitro* model of enterocytes and M cells *via* both induced endocytosis and active penetration.^66^ Whether this invasion translates to translocation across the epithelial barrier, however, remains to be investigated. The contribution of phagocytic cells within Peyer’s patches to the translocation of *C. albicans* has yet to be investigated. However, De Jesus *et al*.^65^ also observed that *C. albicans* is sampled from Peyer’s patches *in vivo* by dendritic cells (Figure 1b). *C. albicans* was also shown to be recognized and internalized by intestinal CX3CR1^+^ mononuclear phagocytes in a mouse model of intestinal colonization.^67^ The fate of these yeast cells has yet to be investigated. It is possible that internalized *C. albicans* cells are able to hijack this mechanism to facilitate their own transport across the intestine in a similar fashion to bacteria. It has been well documented that *C. albicans* is able to survive within phagocytes, filament, damage the host cell, and ultimately escape.^68^ In an *in vivo* zebrafish model of disseminated candidiasis, yeast cells phagocytosed by macrophages were transported away from the infection site through the blood and later escaped.^69^

### 3.3 Paracellular translocation

Though the main route for *C. albicans* translocation is *via* direct invasion and damage of epithelial cells *in vitro*, low levels of translocation could still be observed in a damage-independent manner when using a mutant strain lacking CaL.^59^ As mentioned in section 3.1, this could be the result of invasion and transcellular translocation in the absence of host damage; however, a paracellular route between epithelial cells has also been discussed (Figure 1c).^19^ Enterocytes likely limit fungal translocation to a degree through increased expression of tight and adherens junction proteins during these early stages (Figure 2).^57^ Additionally, the presence of antimicrobial peptides or the quorum sensing molecule farnesol increases the expression of tight junction proteins and consequently improves the epithelial barrier integrity.^70,71^ Nevertheless, the intestinal epithelial barrier shows increased permeability during infection with *C. albicans* even at early time points before significant levels of damage can be detected.^59,60,72,73^ *C. albicans* can degrade tight and adherens junction proteins like E-cadherin, occludin, and demoglein-2.^73^ In fact, *C. albicans* decreases levels of occludin, JAM-A, and claudins 1, 3, 4 starting 6 hours after infection of intestinal epithelial cells with E-cadherin levels dropping after 21 hours.^72^ A similar mechanism can be found in some pathogenic bacteria. *Vibrio cholerae* secretes multiple proteins and toxins that degrade and alter tight junctions.^74^ The secreted haemagglutinin/protease specifically degrades extracellular occludin of host cells.^75^ *V. cholerae* also produces the zonula occludens toxin (ZOT). ZOT reorganizes tight junctions of intestinal epithelial cells *via* interactions with occludin and ZO-1.^76^ Though this rearrangement of tight junctions was not associated with a decrease in the barrier integrity or permeability, *in vivo* experiments have shown that ZOT increases the permeability of the small intestine resulting in a leaky epithelium.^77^

E-cadherin degradation of oral epithelial cells by *C. albicans* has been linked to the secreted aspartyl protease Sap5.^78^ A strain lacking *SAP5*, however, showed no decrease in translocation in an *in vitro* model system of cultured enterocytes.^59,78^ While paracellular translocation is possible and even likely, thus far there is no study with direct evidence that *C. albicans* crosses the intestinal epithelium by degrading tight junctions between enterocytes. Basmaciyan and colleagues^19^ do, however, highlight that disruption of the intestinal barrier and tight junctions by external factors *in vivo* could allow for paracellular translocation *via* mechanisms like the induced endocytosis described in section 3.1.^57^ Further studies are required to elucidate the contribution of the paracellular route to *C. albicans* translocation, the involved fungal factors, and what impact this has under clinically relevant conditions.

## 4. Dissemination *via* the bloodstream

### 4.1 Disseminating phenotype

A unique aspect of *C. albicans* pathogenicity comes into focus when looking at dissemination as compared to bacteria. Most bacteria do not filament and they reach the bloodstream as an easily disseminated shape and size. This is not the case for *C. albicans*. While hyphae are the main invasive form of *C. albicans* during epithelial infection and translocation, yeast cells are thought of as the morphotype best suited for dissemination once the fungus has entered the bloodstream due to their smaller size and rounder shape.^21^ While hyphal and pseudohyphal cells are able to adhere to endothelial cells under flow and shear stress, adhesion of yeast cells was increased under such conditions.^79^ Indeed, yeast with short germ tubes were shown to be the optimal morphology for adhesion to endothelial cells in *in vitro* conditions mimicking physiological flow in the bloodstream.^80^

Distinct roles for both yeast and hyphae have been described in a zebrafish model of disseminated candidiasis.^81^ A hyper-filamentous *C. albicans* strain readily invaded and killed the zebrafish without disseminating through the blood, while hypo-filamentous and yeast-locked strains were able to cause disseminated disease with decreased mortality. When hyper- and hypo-filamentous strains were co-infected there was an additive effect of both morphologies, but no enhancement of virulence.^81^ The same study even observed dissemination of a yeast-locked strain of *C. albicans*. The ability of yeast to exit the bloodstream independently of hypha formation has also been observed in murine models of disseminated candidiasis.^82^ Saville and coworkers^82^ showed that strains locked in the yeast morphology are capable of translocating across the endothelium and colonizing organs of mice to a similar degree as normal filamenting *C. albicans*, though with decreased mortality. Recently, however, it was shown that a *C. albicans* strain lacking the gene *EED1* that is deficient in maintaining hyphal growth is capable of dissemination with no associated drop in virulence.^83^

### 4.2 How do yeast cells reach the bloodstream?

As current studies on *C. albicans* translocation using *in vitro* models suggest, the fungus reaches the bloodstream as long hyphae following continuous invasion and translocation across the intestinal epithelium. Given that yeast cells or short hyphae seem to be better suited for exit from the bloodstream as a prerequisite of organ colonization, the question remains as to how yeast appear in the bloodstream in the first place.^79–82^ Here we explore three different hypotheses that have been proposed to explain the presence of yeast in the bloodstream following translocation: mechanical lesions, a “hitchhiking” mechanism, and a hypha-to-yeast transition (Figure 1e-g).

### Mechanical lesions

Disruption of the intestinal barrier is a major predisposing factor for systemic infection and intestinal translocation in mice colonized with *C. albicans*.^15^ In humans, immunocompromised patients are particularly susceptible to systemic candidemia and often suffer from mucosal damage to the GI tract as a result of chemotherapy treatments.^1,2,84–86^ Given that both yeast and hyphae are present in the GI tract during colonization, physical disruption of the epithelial barrier could allow for the passive translocation of yeasts into the bloodstream.^24–26^ While this is a probable mechanism for patients with disturbed intestinal epithelia, not all individuals at high risk for developing candidemia suffer from this. Critically ill patients in intensive care units are another group that is particularly susceptible to candidemia, though they do not consistently present with mucosal damage due to chemotherapy treatment or abdominal surgery.^1,84–86^ These studies suggest that the development of physical lesions in the epithelium is only responsible for the presence of yeast in the bloodstream for a subset of clinical cases, but it is likely not the only mechanism.

### “Hitchhiking” mechanism

Another possibility is that the yeast present in the GI tract during colonization simply move through the intestinal epithelium together with hyphae as they invade the tissue (Figure 1f). It has been suggested before that adhesins expressed during the later stages of biofilm formation, like Fav2 and Hyr1, may contribute to cell-to-cell adhesion of *C. albicans*.^87^ Loss of *YWP1*, a gene encoding an adhesion protein associated with the yeast morphology of *C. albicans*, decreased the biofilm mass only during the late stages of biofilm development.^88,89^ Additionally, the adhesins encoded by *HWP1* and *ALS3* also interact with each other and *Saccharomyces cerevisiae* yeast cells expressing *C. albicans* Hwp1 were able to bind *C. albicans* hyphae, though only when the hyphae expressed *ALS3*.^90^ Taken together, these *in vitro* studies show that there is the potential for *C. albicans* yeast cells to attach to hyphal cells. However, this has not been showed directly thus far and more studies are required to determine whether this adhesion between morphologies is sufficient to transport yeast cells. It should be noted that a similar mechanism has been proposed for *Candida glabrata* yeast cells attached to *C. albicans* hyphae during oral infections.^91^

A study where immunosuppressed gnotobiotic piglets developed systemic candidiasis following oral inoculation with *C. albicans* showed lesions and extensive necrosis of the intestinal epithelium.^92^ Both yeast and hyphae were observed in the intestinal lesions, which might suggest a “hitchhiking” mechanism where yeast from the intestine move along with translocating hyphae following sufficient damage of host tissue. However, this could also be due to a transition from hyphal to yeast growth during translocation. In an *in vitro* model of intestinal translocation, the damage to host cells caused by invading hyphae is able to degrade the epithelial barrier enough to allow for the passage of dextran particles through.^59^ In a mouse model of colonization and disseminated candidiasis, hyphae and yeast were present invading the intestinal epithelium.^13^ While these studies did not provide direct evidence that yeast cells from the intestinal side cross the epithelium with hyphae, they do provide some support that hyphae are not the only morphotype present during intestinal translocation. These studies and others also show that invading *C. albicans* hyphae produce substantial host damage that allows for material to pass through the barrier, potentially leading to translocation *via* mechanical lesions.^13,14,59^ This degree of epithelial damage during translocation *in vivo* is likely given that there is an expansion of *Candida* spp. just prior to bloodstream infections along with a decrease in the total bacterial burden.^8^ This suggests that both mechanisms, mechanical lesions and “hitchhiking”, may contribute to the presence of yeast cells in the bloodstream given that *C. albicans* reaches a sufficient abundance within the GI tract (Figure 1e, f).

### Hypha-to-yeast transition in human blood

The morphology of *C. albicans* that dominates *in vivo* during growth in the bloodstream is difficult to determine. There are a multitude of different environmental factors like blood cells, serum, flow, and temperature that make finding a simple answer challenging. *C. albicans* seems to readily form filaments during incubation with either serum or whole human blood.^93–96^ Incubation with human serum resulted in over 90% of yeast cells forming hyphae, while incubation with whole blood only resulted in just over 50%.^94^ This difference in morphology was mirrored in a less drastic increase in the expression of hypha-associated genes in whole blood compared to serum. Indeed, during incubation in whole blood *C. albicans* cells mostly associate with neutrophils which overwhelmingly govern the fungal transcriptional response and seem to dampen filamentation.^93^ Over 90% of fungal cells incubated with polymorphonuclear cells were still in the yeast form after 30 minutes. Germ tubes of *C. albicans* incubated in whole blood were also noticeably shorter than those incubated in blood fractions lacking neutrophils.^93^ This agrees with a recent study where only around 20% of counted fungal cells were hyphae following 3 hours of incubation in whole blood.^96^ Though there is evidence that yeast cells are crucial for dissemination through the bloodstream, whether these yeast cells form from invading hyphae originating from the gut has proven more challenging to determine. Understanding what fungal genes are involved in and the conditions that could trigger a switch back to yeast growth is necessary to answer this question.

While the transition from yeast to hypha is well understood, the transition in the opposite direction remains less well defined. This hypha-to-yeast switch, however, is a promising hypothesis and potential mechanism for how yeast reach the bloodstream during translocation (Figure 1g). The transcriptional regulator Pes1 was identified in *C. albicans* to be required for formation of yeast cells from hyphae.^97^ A deletion mutant strain showed decreased formation of lateral yeast from filaments in hypha-inducing conditions but had no effect on filamentous growth itself. *PES1* controls the transition to yeast growth *via* an interaction with Nrg1, a repressor of filamentation in *C. albicans*, during dispersal from biofilms.^98^ These dispersed yeast cells more closely resemble hyphae within the biofilm on a transcriptional level than they do planktonically grown yeast cells. Specifically, they had increased expression of hypha-associated genes (*PGA13, ACE2*), adhesin genes (*ALS5, ALS6*), and secreted aspartyl protease genes (*SAP3*/*6*/*8-9*) among others compared to planktonic cells. *PES1* also shows some function for dissemination during *in vivo* infection. In a jugular-vein catheter mouse model of infection, catheters with biofilms of *PES1*-depleted *C. albicans* showed decreased levels of dissemination and colonization of the kidneys due to impaired lateral yeast formation.^98^

The loss of *PES1* not only decreased *C. albicans* dissemination during systemic infection but attenuated the virulence in a *Galleria mellonella* model of infection.^97^ The contribution of *PES1* to the pathogenicity of *C. albicans* has also been studied in more complex *in vivo* infection models. Uppuluri *et al*.^99^ showed that not only the depletion, but also the overexpression of *PES1* in a mouse model of hematogenously disseminated candidiasis has a negative impact on the virulence of *C. albicans*. Both the overexpression and depletion of *PES1* resulted in decreased mortality during infection. However, overexpression of *PES1* led to a fungal load in organs similar to that of the control and depletion of *PES1* decreased the fungal burden in the brain and kidneys.^99^ The beneficial effect of physiological *PES1* expression during systemic infection highlights the importance of phenotypic plasticity for the pathogenicity of *C. albicans* as well as the likely role of a hypha-to-yeast transition during dissemination (Figure 1g).

## 5. Conclusions and future perspectives

It is clear that *C. albicans* owes much of its success as a commensal and as a pathogen to its morphological plasticity. The contributions of each morphotype to colonization and infection are still not fully understood. This is key to understanding the mechanisms behind intestinal translocation and further dissemination during systemic candidemia. While there has been significant progress in recent years to characterize *C. albicans* translocation *in vitro*, we still lack comprehensive knowledge about how this process is initiated, what it looks like in more complex models or *in vivo* and what happens when the fungus reaches the bloodstream. Further studies are needed to determine which routes *C. albicans* uses to cross the intestinal barrier, the fungal and host factors involved, and how the fungus ultimately spreads throughout the bloodstream to other organs. A better understanding of each of these is important to develop strategies that can ultimately prevent *C. albicans* dissemination and the development of systemic infections.
